# Novel metabolic phenotypes for extrahepatic complication of nonalcoholic fatty liver disease

**DOI:** 10.1097/HC9.0000000000000016

**Published:** 2023-01-10

**Authors:** Jiayi Yi, Lili Wang, Jiajun Guo, Xiangpeng Ren

**Affiliations:** 1 Department of Biochemistry, Medical College, Jiaxing University, Jiaxing, China; 2 Department of Cardiology, Fuwai Hospital, Chinese Academy of Medical Sciences and Peking Union Medical College, National Center for Cardiovascular Diseases, Beijing, China; 3 Department of Cardiology, West China Hospital, Sichuan University, Chengdu, Sichuan Province, China

## Abstract

Phenotypic heterogeneity among patients with NAFLD is poorly understood. We aim to identify clinically important phenotypes within NAFLD patients and assess the long-term outcomes among different phenotypes.

**Methods:**

We analyzed the clinical data of 2311 participants from the Third National Health and Nutrition Examination Survey (NHANES III) and their linked mortality data through December 2019. NAFLD was diagnosed by ultrasonographic evidence of hepatic steatosis without other liver diseases and excess alcohol use. A 2-stage cluster analysis was applied to identify clinical phenotypes. We used Cox proportional hazard models to explore all-cause and cause-specific mortality between clusters.

**Results:**

We identified 3 NAFLD phenotypes. Cluster 1 was characterized by young female patients with better metabolic profiles and lower prevalence of comorbidities; Cluster 2 by obese females with significant insulin resistance, diabetes, inflammation, and advanced fibrosis and Cluster 3 by male patients with hypertension, atherogenic dyslipidemia, and liver and kidney damage. In a median follow-up of 26 years, 989 (42.8%) all-cause mortality occurred. Cluster 1 patients presented the best prognosis, whereas Cluster 2 and 3 had higher risks of all-cause (Cluster 2—adjusted HR: 1.48, 95% CI: 1.16–1.90; Cluster 3—adjusted HR: 1.29, 95% CI: 1.01–1.64) and cardiovascular (Cluster 2—adjusted HR: 2.01, 95% CI: 1.18–3.44; Cluster 3—adjusted HR: 1.75, 95% CI: 1.03–2.97) mortality.

**Conclusions:**

Three phenotypically distinct and clinically meaningful NAFLD subgroups have been identified with different characteristics of metabolic profiles. This study reveals the substantial disease heterogeneity that exists among NAFLD patients and underscores the need for granular assessments to define phenotypes and improve clinical practice.

## INTRODUCTION

NAFLD has a global prevalence of 25% and is becoming a leading cause of end-stage liver disease,^1^ liver cancer,^2^ and even liver-related mortality worldwide.^3^ NAFLD refers to a broad range of clinical and pathological findings and is characterized by remarkable interpatient variability in disease severity and progression.^4^,^5^ The primary driving factors of disease can vary substantially among patients with NAFLD.^6^ Therefore, the population of NAFLD patients is considered heterogeneous.^7^

NAFLD is recognized as the liver component of a collection of conditions that are associated with metabolic dysfunction, and the mechanisms by which liver and global metabolic derangement contribute are complex and heterogeneous.^8^ In 2020, a new conception, namely metabolic dysfunction-associated fatty liver disease (MAFLD), was proposed by an international panel of experts to highlight the role of metabolic risk factors in the development and progression of liver disease.^9^ However, the utility of this term is still debatable.^6^,^10^,^11^ In addition, the definition of metabolic dysfunction in MAFLD is also a complex syndrome, and heterogeneous might exist within this population. Therefore, the clinical manifestations and natural history of different subtypes of NAFLD remain poorly understood.^12^

Precise phenotyping is essential to identify the disease severity and provide prognostic information for patients with NAFLD. Cluster analysis, an unsupervised machine learning technique, has been extensively used in identifying phenotypes in various diseases.^13^–^15^ It can lead to improved characterization of disease phenotypes with different natural histories, outcomes, and potentially therapeutic responses.^16^ The aim of this study is to apply cluster analysis to identify clinically important phenotypes within a population-based cohort of NAFLD patients in the United States and assess the long-term outcomes among derived patient clusters.

## METHODS

### Study design and population

From 1988 to 1994, National Health and Nutrition Survey (NHANES) III was conducted by the National Center for Health Statistics (NCHS). It aims to assess health and nutritional status using a nationally representative sample of noninstitutionalized US civilians. The study protocol was approved by the institutional review board of the NCHS. Written informed consent to participate in NHANES III was obtained from all participants. This study was reviewed by the Fuwai institutional review board and considered exempt.

Of the 20,050 adult participants from NHANES III, 14,797 participants aged 20–74 years underwent hepatic ultrasound examination. We excluded 11,644 participants without ultrasonographic identified hepatic steatosis. A total of 296 were further excluded due to viral hepatitis (positive serum hepatitis C antibody and/or positive serum hepatitis B surface antigen), and 141 were excluded due to excessive alcohol use (>2 or 3 standard alcoholic drinks per day on average for women or men, respectively). One participant lost to follow-up was additionally excluded. Finally, we excluded 404 participants with missing data of clustering variables. The final study cohort included 2311 NAFLD patients (Supplementary Figure 1, http://links.lww.com/HC9/A39).

### Clinical and laboratory measurements

The detailed descriptions of demographical, anthropometric, and laboratory variables have been previously described.^17^,^18^ In this study, the average alcohol consumption per day was calculated using the frequency and amount of alcohol consumed per drinking day. Current smoker was defined as those who reported ongoing smoking and had smoked more than 100 cigarettes lifetime. We defined hypertension by self-report history or the objective measurements (with mean systolic blood pressure ≥140 mm Hg or diastolic blood pressure ≥90 mm Hg over 3 consecutive blood pressure measurements). Diabetes was defined as having a self-reported history or having hemoglobin A1c ≥6.5%. Insulin resistance was assessed using the homeostatic model assessment-insulin resistance (HOMA-IR) equation^19^ and was defined as having HOMA-IR>2.5. The estimated glomerular filtration rate (eGFR) was calculated using the Modification of Diet in Renal Disease formula.^20^

### Definition of NAFLD and advanced fibrosis

The method used for the gallbladder/hepatic ultrasound-diagnosed fatty liver in NHANES III has been described previously.^10^,^21^ Briefly, the hepatic ultrasonography was conducted using a Toshiba SSA-90A ultrasound machine (Tustin, CA) in adult participants (aged 20–74 y). The archived gallbladder ultrasound examination videotapes were reviewed by 3 certified radiologists between 2009 and 2010 to grade the presence of fat within the hepatic parenchyma under 5 criteria. The ultrasonographic findings were reported as normal versus mild, moderate or severe hepatic steatosis.^22^ The intrarater and inter-rater reliability (percent agreement) of the assessments were 91.3% and 88.7%, respectively.^10^ In this study, NAFLD was defined by having moderate or severe hepatic steatosis determined by ultrasonography in the absence of excessive alcohol consumption and other causes of chronic liver disease. Patients with at least 1 of the 3 noninvasive liver fibrosis scores (NAFLD fibrosis score,^23^ fibrosis-4,^24^ and aspartate aminotransferase to platelet ratio index^25^) indicated a high probability of advanced fibrosis (NAFLD fibrosis score >0.676 or fibrosis-4 >2.67 or aminotransferase to platelet ratio index >1.5) were deemed to have advanced liver fibrosis.^11^

### Mortality ascertainment

The mortality data of NHANES III adult participants (≥20 y) were obtained from the National Death Index up to December 2019. The underlying cause of death 113 (UCOD_113) code was encoded by the definition of the International Classification of Diseases, 9th Edition for deaths through 1998, and the International Classification of Diseases, 10th Edition for deaths from 1999 to 2015. Cause-specific mortalities were considered, including cardiovascular disease (UCOD_113 code: 55-64, 70) and cancer (UCOD_113 code: 19-43).^11^ The duration of follow-up was defined as the interval from the interview date to the date of death or through December 31, 2019, for participants without event.

### Cluster analysis

We selected 21 baseline variables to represent clinically significant and prevalent features and comorbidities in the NAFLD population (Supplementary Table 1, http://links.lww.com/HC9/A39). A 2-stage cluster analysis was performed to determine the clusters: (1) A hierarchical cluster analysis using Ward’s method applying Euclidean distance was used to determine the optimum number of clusters after variable standardization. The number of clusters was restrained within 12 clusters and objectively evaluated by iteratively repeating clustering under 24 performance measures indices.^26^ The number voted by most indices was determined as the optimal number of clusters; (2) We then reran the hierarchical cluster analysis with the optimal number of clusters to allocate each patient into a particular cluster. Hierarchical dendrograms were plotted to illustrate the linkage between patients at increasing levels of dissimilarity, which allows us to identify how the clusters are merged. The Boruta analysis was applied to find the relative importance of the variables in predicting cluster membership.^27^ We further used *Z* scores of variables calculated during variable standardization in different clusters to identify the feature of clusters.

### External replication

An external replication of the cluster analysis was conducted using data from NHANES 2017 to March 2020. The study population and variable definitions of NHANES 2017–March 2020 were described in Supplementary Methods, http://links.lww.com/HC9/A39. The same clustering technique used in NHANES III was also applied to the external replication data.

### Statistical analysis

The means [SD)/median (interquartile range)] for normally/skewed distributed continuous variables and counts (percentage) of categorical variables were presented among different clusters. Baseline characteristics were compared using the *t* test/standard nonparametric tests for normally/skewed distributed continuous variables and the χ^2^ test for categorical variables. We used Cox proportional hazards models to determine HRs and 95% CIs for the associations between NAFLD clusters and risks of all-cause and cause-specific mortality in the NHANES III cohort. Two multivariable models were constructed. In model 1, we adjusted for age and sex. In model 2, race/ethnicity and body mass index (BMI) were additionally adjusted. The proportional hazards assumption was examined by creating time-dependent covariates in the models.^28^ No apparent violation of the proportional hazards assumption was identified (*p*>0.05). Survival of all-cause mortality among clusters was also evaluated using Kaplan-Meier curves. Statistical tests were 2-sided, and statistical significance was set at *p*<0.05. All statistical analyses were conducted using R software, version 4.2.0 (R Core Team, Vienna, Austria).

## RESULTS

**FIGURE 1:**
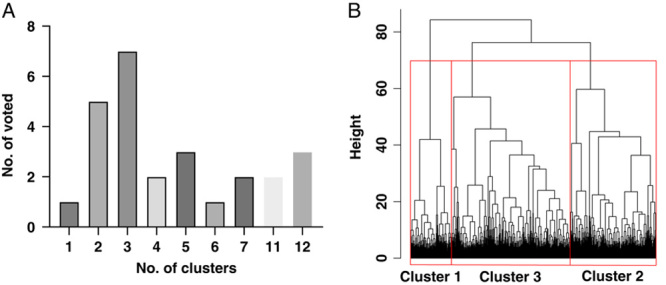
The bar plot determines the optimum number of clusters (A) and dendrogram of the final hierarchical clustering model (B). Wald’s minimum-variance hierarchical clustering method and the bottom-up approach were used. All subjects were clustered into a single final group. At each generation of clusters, samples were merged into larger clusters to minimize the within-cluster sum of squares or maximize the between-cluster sum of squares. With successive clustering, 3 groups became obvious. Abbreviations: NHANES indicates National Health and Nutrition Examination Surveys.

**FIGURE 2:**
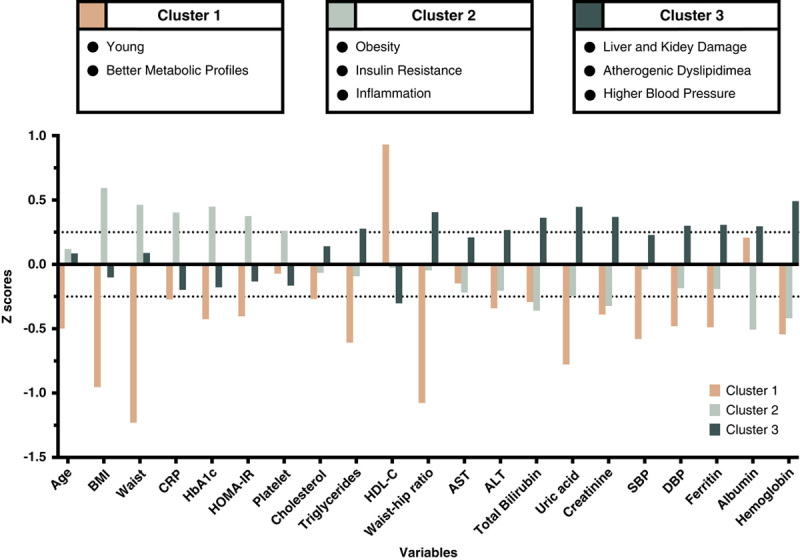
Bar plot of the cluster profiles using clustering variables. The *Z* score of the variables has been used to standardize each variable. Values above 0.25 indicate those variables able to phenotype the 3 clusters. Abbreviations: ALT indicates alanine aminotransferase; AST, aspartate aminotransferase; BMI, body mass index; CRP, C-reactive protein; DBP, diastolic blood pressure; HbA1c, glycohemoglobin; HDL-C high-density lipoprotein cholesterol; HOMA-IR, homeostatic model assessment-insulin resistance; SBP, systolic blood pressure.

Three distinct clusters were identified. (Figure 1). Among 21 variables used in cluster analysis, BMI, waist circumference, hemoglobin, and glycohemoglobin were identified as the most important variables in the prediction of the patient cluster (Supplementary Figure 2, http://links.lww.com/HC9/A39). To highlight the major differences between clusters, Figure 2 presents bar plots of *Z* scores of each variable within each cluster. The baseline characteristics of the study population grouped by identified clusters are summarized in Table 1.

**TABLE 1:** Baseline characteristics of the study population

	Overall (n=2311)	Cluster 1 (n=385)	Cluster 2 (n=810)	Cluster 3 (n=1116)	*p*
Age (y)	47.7 (15.3)	40.0 (15.8)	49.5 (14.4)	49.0 (15.0)	<0.01
Female, n (%)	1174 (50.8)	293 (76.1)	608 (75.1)	273 (24.5)	<0.01
Race/ethnicity, n (%)					<0.01
Non-Hispanic White	854 (37.0)	121 (31.4)	288 (35.6)	445 (39.9)	
Non-Hispanic Black	450 (19.5)	113 (29.4)	186 (23.0)	151 (13.5)	
Mexican American	929 (40.2)	141 (36.6)	305 (37.7)	483 (43.3)	
Other	78 (3.4)	10 (2.6)	31 (3.8)	37 (3.3)	
Health status, n (%)					<0.01
Excellent or good	1640 (71.0)	293 (76.1)	521 (64.3)	826 (74.0)	
Fair	545 (23.6)	84 (21.8)	219 (27.0)	242 (21.7)	
Poor	125 (5.4)	8 (2.1)	69 (8.5)	48 (4.3)	
Current smoker, n (%)	483 (20.9)	95 (24.7)	135 (16.7)	253 (22.7)	<0.01
Hypertension, n (%)	1079 (46.7)	83 (21.6)	402 (49.6)	594 (53.2)	<0.01
SBP (mm Hg)	130.5 (20.0)	118.9 (18.3)	129.7 (17.6)	135.1 (20.5)	<0.01
DBP (mm Hg)	78.7 (11.5)	73.2 (10.2)	76.6 (10.7)	82.1 (11.4)	<0.01
Waist circumference (cm)	101.9 (15.1)	83.3 (10.7)	108.9 (14.9)	103.3 (10.8)	<0.01
Waist-hip ratio	1.0 (0.1)	0.9 (0.1)	1.0 (0.1)	1.0 (0.1)	<0.01
BMI (kg/m^2^)	30.4 (6.5)	24.3 (4.0)	34.3 (7.3)	29.8 (4.3)	<0.01
Diabetes, n (%)	462 (20.0)	20 (5.2)	274 (33.8)	168 (15.1)	<0.01
Insulin resistance, n (%)	1578 (68.3)	102 (26.5)	670 (82.7)	806 (72.2)	<0.01
HbA1c, n (%)	5.9 (1.5)	5.3 (0.8)	6.6 (2.1)	5.7 (1.0)	<0.01
Fasting glucose (mg/dL)	113.3 (53.1)	94.0 (30.1)	133.8 (76.1)	105.0 (28.7)	<0.01
Hemoglobin (g/dL)	14.2 (1.5)	13.4 (1.1)	13.6 (1.5)	14.9 (1.3)	<0.01
Platelet counts (1000 cells/µL)	279.6 (72.0)	274.5 (66.2)	298.4 (78.2)	267.8 (66.2)	<0.01
Ferritin, (ng/mL)	117.0 [55.5, 219.0]	54.0 [27.0, 110.0]	91.0 [36.2, 185.8]	168.0 [93.0, 269.0]	<0.01
CRP (mg/dL)	0.2 [0.2, 0.6]	0.2 [0.2, 0.2]	0.5 [0.2, 1.0]	0.2 [0.2, 0.4]	<0.01
Cholesterol (mg/dL)	212.5 (44.2)	200.6 (45.0)	209.6 (42.0)	218.7 (44.5)	<0.01
Triglycerides (mg/dL)	156.0 [104.5, 235.0]	92.0 [67.0, 135.0]	157.5 [113.0, 218.0]	181.5 [125.0, 281.0]	<0.01
HDL-C (mg/dL)	46.12 (14.9)	60.0 (19.8)	45.7 (12.2)	41.6 (11.5)	<0.01
Total Bilirubin (mg/dL)	0.6 (0.3)	0.5 (0.2)	0.5 (0.2)	0.7 (0.4)	<0.01
AST (U/L)	21.0 [17.0, 28.0]	19.0 [17.0, 25.0]	19.0 [15.2, 24.0]	24.0 [19.0, 30.0]	<0.01
ALT(U/L)	19.0 [13.0, 28.0]	14.0 [10.0, 19.0]	17.0 [12.0, 23.0]	22.0 [16.0, 34.0]	<0.01
Albumin (g/dL)	4.1 (0.4)	4.2 (0.4)	4.0 (0.3)	4.3 (0.3)	<0.01
Uric acid (mg/dL)	5.7 (1.5)	4.5 (1.1)	5.4 (1.4)	6.4 (1.4)	<0.01
Creatinine (mg/dL)	1.1 (0.3)	1.0 (0.2)	1.0 (0.2)	1.2 (0.3)	<0.01
eGFR (ml/min/1.73m^2^)	73.6 (15.9)	78.6 (15.7)	74.1 (15.9)	71.5 (15.5)	<0.01
Advanced fibrosis, n (%)	118 (5.1)	9 (2.3)	60 (7.4)	49 (4.4)	<0.01

Values were displayed as mean (SD) or median [interquartile range] for continuous variable and count (%) for categorical variable.

Abbreviations: ALT indicates alanine aminotransferase; AST, aspartate aminotransferase; BMI, body mass index; CRP, C-reactive protein; DBP, diastolic blood pressure; eGFR, estimated glomerular filtration rate; HbA1c, glycohemoglobin; HDL-C high-density lipoprotein cholesterol; SBP, systolic blood pressure

### Baseline characteristics

Cluster 1 describes a phenotype of younger females with better metabolic profiles and a lower prevalence of comorbidities. This cluster includes the highest percentage of females (76.1%) with the lowest mean age of 40.0 years. The percentage of the current smoker is higher in Cluster 1 (24.7%), and the prevalence of diabetes and hypertension is lower (5.2% and 21.6%, respectively). 76.1% of patients in this cluster reported excellent or good health status. This cluster also presents better metabolic profiles with the lowest BMI, waist circumference, blood pressure, liver enzymes, glycolipid metabolism and inflammation biomarkers, and highest eGFR. Advanced hepatic fibrosis is also less common in Cluster 1 (2.3%).

Cluster 2 describes a phenotype of obese females with significant insulin resistance, diabetes, inflammation, and advanced fibrosis. The mean age in this cluster is 49.5 years, and the proportion of females is as high as Cluster 1 (75.1%). Only 64.3% of patients in this cluster reported excellent or good health status. The proportion of smokers is the lowest in this cluster (16.7%). The waist circumference and BMI are both highest in Cluster 2, as well as the prevalence of diabetes (33.8%). 82.7% of patients in Cluster 2 have insulin resistance. The levels of fasting glucose and C-reactive protein are also highest in these patients compared with the other 2 clusters. Of note, the prevalence of advanced liver fibrosis is also highest in Cluster 2 (7.4%).

Cluster 3 describes a phenotype of males with significant hypertension, atherogenic dyslipidemia, and liver and kidney damage. The average age of patients in Cluster 3 is 49.0 years. Only 24.5% of patients in this cluster are female. The blood pressure levels (mean systolic blood pressure 135.1 mm Hg; mean diastolic blood pressure 82.1 mm Hg) and the prevalence of hypertension (53.2%) are highest in this cluster. Although the prevalence of diabetes is lower than in Cluster 2 (15.1% vs. 33.8%), 72.2% of patients in Cluster 3 are identified as having insulin resistance. Cluster 3 also presents with atherogenic dyslipidemia (higher cholesterol, triglycerides, and lower high-density lipoprotein cholesterol) higher liver enzymes (aspartate aminotransferase and alanine aminotransferase), total bilirubin, uric acid, and creatinine. The eGFR is the lowest in this cluster as well.

### Cluster association with clinical outcomes

**FIGURE 3:**
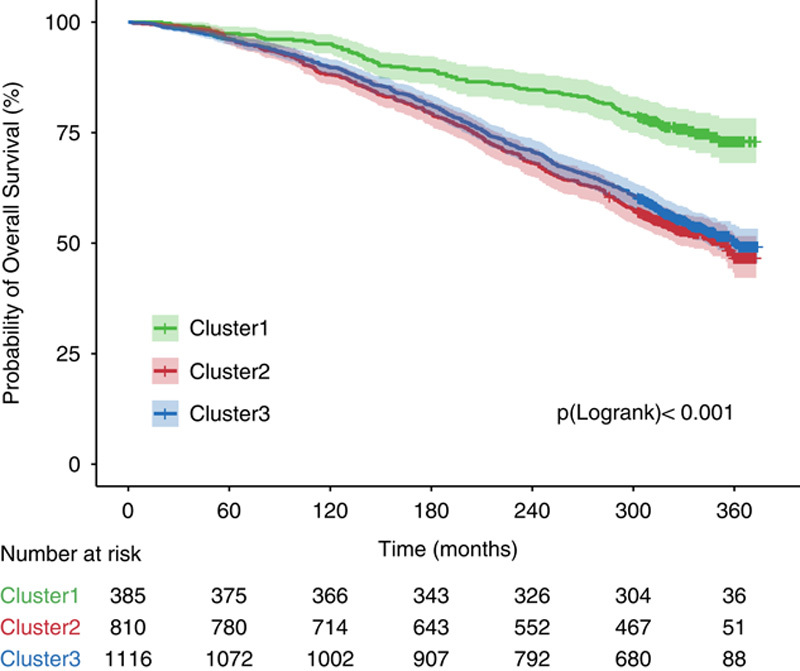
Kaplan-Meier survival curves for all-cause mortality of the 3 clusters of NAFLD patients. Pairwise Log-Rank test: Cluster 1 versus Cluster 2, *p*<0.001; Cluster 1 versus Cluster 3, *p*<0.001; Cluster 2 versus Cluster 3, *p*=0.26.

During a median follow-up period of 312 months (up to 374 mo), 989 (42.8%) all-cause deaths occurred. Among them, 274 (27.7%) deaths were cardiovascular related, and 215 (21.7%) deaths were cancer related. The rates of all-cause mortality were 9.9, 21.6, and 20.2 per 1000 person-years for 3 clusters separately (Table 2). The cox model showed that compared to patients in Cluster 1, individuals in Cluster 2 and Cluster 3 were all associated with higher all-cause mortality risk. After the adjustment of potential confounders, the elevated risks of all-cause mortality remain significant in Cluster 2 (adjusted HR: 1.48; 95% CI: 1.16–1.90) and Cluster 3 (adjusted HR: 1.29; 95% CI: 1.01–1.64). Adjusted HRs for cardiovascular mortality among Cluster 2 and Cluster 3 compared with Cluster 1 were 2.01 (95% CI: 1.18–3.44) and 1.75 (95% CI: 1.03–2.97) separately. As for cancer mortality, Cluster 2 had an 84% higher risk (HR: 1.84; 95% CI:, 1.18–2.88), and Cluster 3 had a 68% higher risk (HR: 1.68; 95% CI: 1.09–2.60) than Cluster 1 (Table 2). The difference in risk for cancer mortality among the 3 clusters attenuated and became insignificant after adjusting for potential confounders. Kaplan-Meier curves show that Cluster 2 and Cluster 3 have similar rates of all-cause mortality (*p* for pairwise log-rank=0.26), which were both significantly higher than in Cluster 1 (*p* for pairwise log-rank <0.001) (Figure 3).

**TABLE 2:** Association between NAFLD clusters and all-cause and cause-specific mortality

			Unadjusted Model	Multivariable Model 1^a^			Multivariable Model 2^b^	
	Deaths, n	Weighted Death (%)	HR (95% CI)	*p*	HR (95% CI)	*p*	HR (95% CI)	*p*
All-cause mortality								
Cluster 1	95	9.9	1 (Reference)	—	1 (Reference)	—	1 (Reference)	—
Cluster 2	386	21.6	2.29 (1.83–2.87)	<0.01	1.48 (1.18–1.85)	<0.01	1.48 (1.16–1.90)	<0.01
Cluster 3	508	20.2	2.13 (1.71–2.65)	<0.01	1.26 (1.00–1.56)	0.05	1.29 (1.01–1.64)	0.04
Cardiovascular mortality								
Cluster 1	18	1.9	1 (Reference)	—	1 (Reference)	—	1 (Reference)	—
Cluster 2	108	6.0	3.33 (2.30–5.49)	<0.01	2.08 (1.26–3.43)	<0.01	2.01 (1.18–3.44)	0.01
Cluster 3	148	5.9	3.23 (1.98–5.26)	<0.01	1.75 (1.05–2.92)	0.03	1.75 (1.03–2.97)	0.04
Cancer mortality								
Cluster 1	25	2.6	1 (Reference)	—	1 (Reference)	—	1 (Reference)	—
Cluster 2	83	4.6	1.84 (1.18–2.88)	<0.01	1.19 (0.76–1.87)	0.45	1.19 (0.72–1.97)	0.49
Cluster 3	107	4.3	1.68 (1.09–2.60)	0.02	0.97 (0.60–1.54)	0.88	0.97 (0.59–1.58)	0.90

^a^ Adjusted for age and sex.

^b^ Additionally adjusted for race/ethnicity and BMI.

Abbreviations: BMI indicates body mass index.

### External replication in the NHANES 2017-2020

Using data from NHANES 2017 to March 2020, 3 clusters were also identified with similar baseline demographic profiles as NHANES III (Supplementary Figure 3, http://links.lww.com/HC9/A39; Supplementary Table 2, http://links.lww.com/HC9/A39). Aligned with the NHANES III data, Cluster 1 describes a phenotype with better metabolic profiles and a lower prevalence of comorbidities; Cluster 2 describes a phenotype of obese female patients with significant insulin resistance, inflammation, and advanced hepatic fibrosis, while Cluster 3 describes a phenotype of older male patients with significant hypertension, atherogenic dyslipidemia, and liver and kidney damage. Of note, NASH identified by the FibroScan-AST score was most prevalent in Cluster 3 (21.7%). Different from NHANES III, the age gaps among the 3 clusters are smaller. In addition, the prevalence of diabetes was higher in Cluster 3 (40.6%) than in Cluster 2 (36.6%).

## DISCUSSION

In this study, we applied cluster analysis, an exploratory data-mining technique, in the NHANES III dataset to identify clinical phenotypes within NAFLD patients. We report the following major findings: (1) 3 distinct clusters were identified in NAFLD patients and had clinically different phenotypes; (2) these clusters were associated with different risks of long-term clinical outcomes; (3) the profiles of the clusters were externally replicated using data from NHANES 2017 to March 2020.

Clustering techniques have been extensively applied in phenotype mapping of several heterogeneous clinical syndromes, such as asthma, idiopathic pulmonary arterial hypertension, and heart failure,^16^,^29^–^31^ which provided key insights into disease pathophysiology and clinical practice. Recently, a study identified 5 clusters of MAFLD from a Chinese cohort using cluster analysis and validated the results in a UK cohort.^32^ Patients in different clusters exhibited different risks of extrahepatic complication and all-cause mortality. However, the study only used a limited number of variables for clustering and the analysis of the difference in health outcomes among clusters was not adjusted for confounders. Our results highlight the significant clinical heterogeneity within patients with NAFLD and the need for novel multidimensional NAFLD phenotyping for improved patient care.

Describing differences in patterns of clinical presentation could improve our understanding of the pathophysiology process and disease heterogeneity. Until now, 3 major NAFLD-inducing pathways have been identified: (1) hepatic genetic component, (2) adipose tissue dysfunction component, and (3) hepatic de-novo lipogenesis component.^12^ However, it is not easy to distinguish these components in NAFLD patients in clinical practice for the scarcity of clinical data. We identified 3 distinct clusters of patients who differ in demographical, anthropometrical, and metabolic parameters and long-term prognosis in this study. Results from this study might provide insights into this issue.

Cluster 1 reflected a group of younger female patients with better metabolic profiles and excellent long-term survival. This phenotype may correspond to the hepatic genetic pathway, which is associated with protective effects from cardiovascular disease and the absence of insulin resistance in the NAFLD patient.^12^ Genome-wide association and exome sequencing studies derived robust evidence that although the genetic variability in PNPLA3, TM6SF2, MBOAT7, GCKR, and HSD17B13 are associated with increased susceptibility and progression of NAFLD,^12^ these variants are also unexpectedly associated with apparent protection effects from cardiovascular disease such as lowing blood triglycerides, low-density lipoprotein cholesterol concentration, and protection from coronary artery disease.^7^ The lipid profiles and the absence of insulin resistance of patients in Cluster 1 fit the features of the hepatic genetic components.

Cluster 2 represents a group of female obese patients with significant insulin resistance, diabetes, inflammation, advanced fibrosis, and poor prognoses. Phenotypes of Cluster 2 are in accordance with the evolution of hepatic steatosis in obesity which was mainly driven by adipose dysfunction. Obesity is correlated with the expansion of adipose tissue, which leads to dysfunction and death of adipocytes. In the setting of adipose dysfunction, macrophages infiltrate into the adipose tissue and induce inflammation that promotes insulin resistance.^6^ In the context of insulin resistance, inappropriate lipolysis and the compromised fat-storing ability of adipose tissue result in the release of free fatty acids into the circulation, which then becomes available for uptake by the liver and overwhelms its metabolic capacity.^6^,^33^,^34^ Hepatic de-novo lipogenesis might also contribute the pathophysiology of this cluster. Hyperglycemia and hyperinsulinemia were recognized as the main drives of hepatic de-novo lipogenesis.^35^,^36^ Among 3 clusters, patients in Cluster 2 present with the highest fasting glucose levels and HOMA-IR levels, which might induce the lipogenesis process which accelerates the lipid accumulation in the liver. Inflammation led by adipose dysfunction is associated with the initiation and progression of fibrosis which could explain the highest prevalence of advanced fibrosis in Cluster 2.^33^

Cluster 3 describes a group of older male patients with a higher prevalence of atherogenic dyslipidemia, hypertension, and liver and kidney damage. In addition, based on results from external replication, the prevalence of NASH was also highest in this cluster. The liver is a major site of lipid metabolism in the form of the combination of de-novo lipogenesis as well as uptake and secretion of serum lipoproteins.^37^ With the highest level of atherogenic lipids, especially triglycerides, we hypothesize that hepatic de-novo lipogenesis is the main facilitating factor of the pathophysiology of this cluster. The increased intake of dietary fructose is also a strong stimulator of hepatic de-novo lipogenesis.^12^ In addition, excessive fructose intake was also associated with an increased risk of NASH, hyperuricemia, as well as chronic kidney disease.^38^ Therefore, dietary factors might also play an important role in the metabolic disorders in Cluster 3. Atherogenic dyslipidemia is correlated with an increased risk of atherosclerotic vascular disease. The potential for renal endothelial dysfunction and renovascular damage also exists in patients with atherogenic dyslipidemia,^39^ which could explain the higher prevalence of hypertension and impaired kidney function in Cluster 3. Phenotypes of Cluster 3 are in accordance with the growing evidence that NAFLD, especially NASH form, exacerbates predisposes to atherogenic dyslipidemia and releases a variety of procoagulant, thrombogenic, and profibrogenic factors that may promote the development of cardiovascular disease, chronic kidney disease, diabetes, and other extrahepatic chronic diseases.^40^

Understanding disease heterogeneity can be helpful to develop pathophysiological-based prevention and treatment approaches. For patients in Cluster 1, further evaluation of the patients in this cluster is desirable in the context of research studies is appropriate since there is no obvious metabolic disorder pattern presented in these patients. Patients in Cluster 2 were most likely driven by adipose tissue dysfunction, and treatment improves adipocyte insulin resistance and promotes preadipocyte differentiation in subcutaneous adipose tissue could be considered.^34^ Patients in Cluster 3 with significant dyslipidemia and hypertension should implement specific treatment of the cardiometabolic risk parameters early on.

There are several limitations of this study. First, we are unable to dynamic evaluate longitudinal changes in clinical features of the study population since the NHANES III dataset does not provide follow-up examinations. Second, advanced fibrosis and NASH in this study were evaluated by noninvasive assessment tools. However, it is neither practical nor feasible to perform liver biopsies on a vast population for its well-known limitations. These tools could be regarded as satisfactory substitutions for liver biopsy in the setting of a large population-based study. Third, we were unable to further explore the difference in liver-related mortality among the three clusters since the NCHS restricts this information for public usage. Access to these restricted data could provide valuable knowledge in the context of NAFLD phenotypes and liver-related deaths. Finally, as discussed above, genetic and epigenetic factors might further explain the substantial interindividual variation in phenotype, severity, and progression of NAFLD, while our study did not include genetic data of the study population for the data restriction. Further studies considering both clinical and genetic data were needed.

In conclusion, this study indicates that by using a clustering algorithm on clinically available data of patients with NAFLD, we can identify 3 phenotypically distinct and clinically meaningful subgroups. We have also shown that patients in each cluster presented different outcomes on long-term follow-up. These findings highlight the significant heterogeneity that exists among patients with NAFLD and the need for improved advanced phenotyping with multimodal data to facilitate further evaluation.

## Supplementary Material
