# Supplementary material for: Novel metabolic phenotypes for extrahepatic complication of nonalcoholic fatty liver disease
Source: Hepatol Commun. 2023 Jan 10;7(1):e0016. doi: 10.1097/HC9.0000000000000016 (PMC9833442; doi:10.1097/HC9.0000000000000016)
Supplement: Supplementary file 1 [file hc9-7-e0016-s001.docx]

**SUPPLEMENTARY MATERIALS**

**Novel Metabolic Phenotypes for Extrahepatic Complication of Non-alcoholic Fatty Liver Disease in the US: Results from NHANES**

Jiayi Yi, Lili Wang, Jiajun Guo, Xiangpeng Ren

**Table of Contents**

Supplementary Methods for NHANES 2017-March 20202

Supplementary Figure 15

Supplementary Table 16

Supplementary Figure 27

Supplementary Figure 38

Supplementary Table 29

**Supplementary Methods for NHANES 2017** **-March 2020**

***Study design and population***

The National Health and Nutrition Examination Surveys (NHANES) have been conducted on 2-year cycles since 1999 to monitor the health and nutritional status of the US population. As a result of the coronavirus disease 2019 (COVID-19) pandemic, the NHANES program was suspended in March 2020. Data collection for the NHANES 2019-2020 cycle was not completed. Therefore, data collected from 2019 to March 2020 were combined with data from the NHANES 2017-2018 cycle to form a nationally representative sample of NHANES 2017-March 2020 pre-pandemic data. The NCHS ethics review board has approved the NHANES protocols. Written informed consent was obtained from all participants before completing the survey.

In the 2017-March 2020 cycle, 15560 participants were enrolled. Among them, 9021 individuals have conducted vibration-controlled transient elastography (VCTE). Individuals were excluded if they had one of the following conditions: (1) aged less than 20 years (n = 1626); (2) having hepatitis B or C history (n = 221) (2) consuming more than 2 or 3 standard alcoholic drinks per day on average for both women and men, respectively (n = 214), (3) without significant VCTE measured fatty liver (n = 4374), (4) having missing values for clustering variables (n = 1504), leaving a total of 1082 participants for the external replication analysis.

***Baseline Characteristics***

The detailed descriptions of demographical, anthropometric, and laboratory variables have been previously described.^1,2^ The definitions of average alcohol consumption, smoking, hypertension, diabetes, insulin resistance, and estimated glomerular filtration rate were the same as the NHANE III.

***Definition of NAFLD,*** ***Nonalcoholic Steatohepatitis (NASH), and advanced fibrosis***

Transient elastography examinations for all participants aged 12 years and older in NHANES 2017–March 2020 cycle. Participants were examined to assess the controlled attenuation parameter (CAP) score and liver stiffness measurements using the FibroScan^®^ model 502 V2 Touch (Echosens, Waltham, MA). A detailed protocol of transient elastography examinations has been published previously.^1^ In brief, a complete examination was defined as 10 or more valid stiffness measurements, fasting time of at least 3 hours, and liver stiffness interquartile range/median ≤ 30%. According to the literature, the median CAP was dichotomized using 285 dB/m as a threshold for liver steatosis diagnosis with optimum diagnostic performance (sensitivity of 80% and specificity of 77%).^3^ Advanced fibrosis was determined using FibroScan^®^ measured liver stiffness. Patients were considered to have advanced fibrosis with liver stiffness ≥13.1 kPa.^4^ FibroScan-AST (FAST) score was applied to evaluate the presence of nonalcoholic steatohepatitis (NASH).^5^ Patients with FAST score≥0.35 (sensitivity 90%) were considered having NASH. ^1^

**Reference**

1. Vilar-Gomez E, Vuppalanchi R, Mladenovic A, et al. Prevalence of High-risk Nonalcoholic Steatohepatitis (NASH) in the United States: Results From NHANES 2017-2018. *Clin Gastroenterol Hepatol* 2021.

2. Unalp-Arida A, Ruhl CE. Transient Elastography-Assessed Hepatic Steatosis and Fibrosis Are Associated With Body Composition in the United States. *Clin Gastroenterol Hepatol* 2022; **20**(4): e808-e30.

3. Siddiqui MS, Vuppalanchi R, Van Natta ML, et al. Vibration-Controlled Transient Elastography to Assess Fibrosis and Steatosis in Patients With Nonalcoholic Fatty Liver Disease. *Clin Gastroenterol Hepatol* 2019; **17**(1): 156-63.e2.

4. Kim D, Cholankeril G, Loomba R, Ahmed A. Prevalence of Fatty Liver Disease and Fibrosis Detected by Transient Elastography in Adults in the United States, 2017-2018. *Clin Gastroenterol Hepatol* 2021; **19**(7): 1499-501.e2.

5. Newsome PN, Sasso M, Deeks JJ, et al. FibroScan-AST (FAST) score for the non-invasive identification of patients with non-alcoholic steatohepatitis with significant activity and fibrosis: a prospective derivation and global validation study. *Lancet Gastroenterol Hepatol* 2020; **5**(4): 362-73.


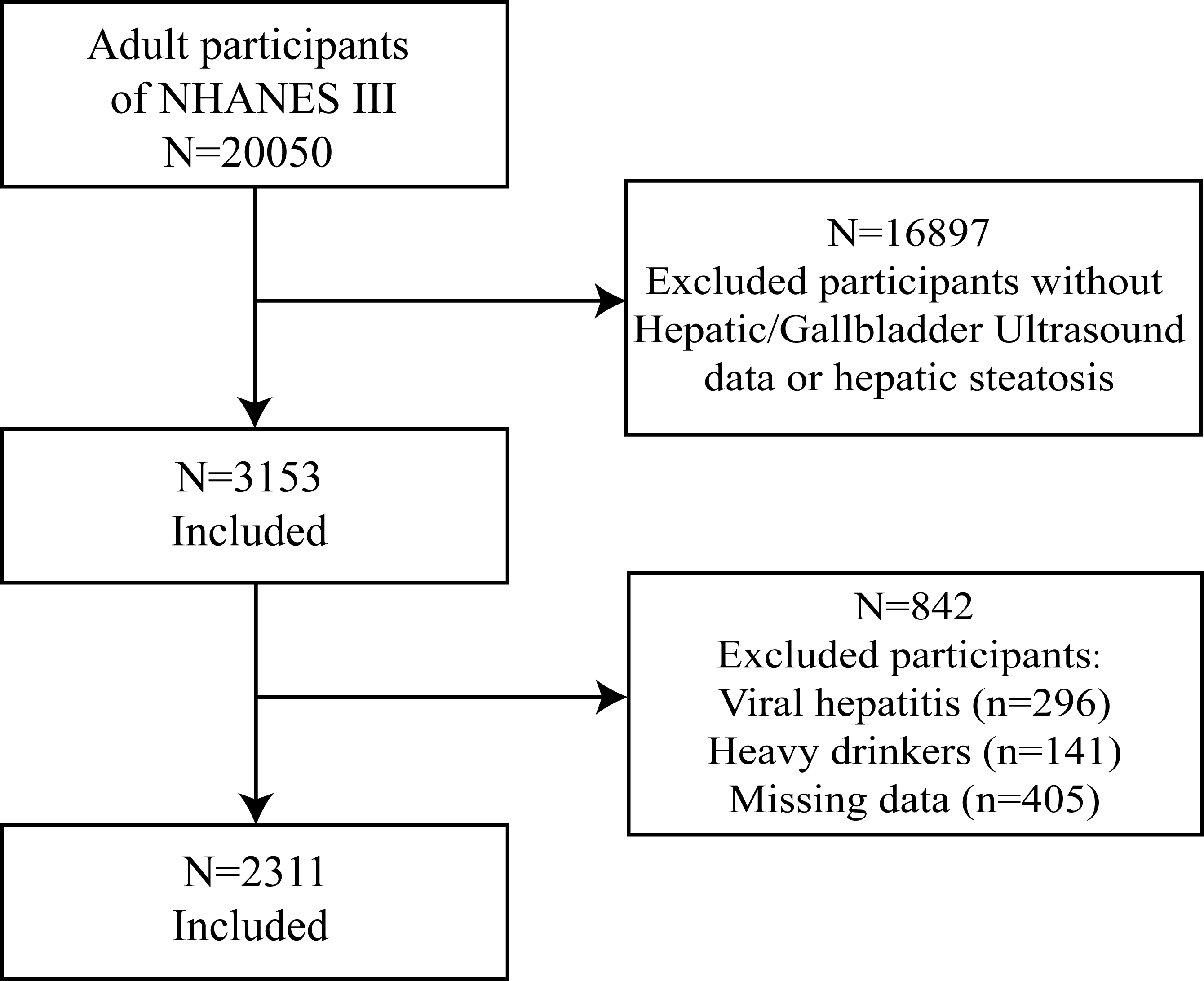


**Supplementary Figure 1. Flow chart of the screening process for the selection of the study population in NHANES III.**

Abbreviations: NHANES, national health and nutrition examination surveys.

**Supplementary Table 1. Clustering variables list.**

| **Category** | **Variable name** |
| --- | --- |
| **Demographics Data** |  |
|  | Age |
| **Examination Data** |  |
|  | Body Mass Index |
|  | Waist Circumference |
|  | Waist-to-Hip Ratio |
|  | Diastolic Blood Pressure |
|  | Systolic Blood Pressure |
| **Laboratory Data** |  |
|  | Total Cholesterol |
|  | Triglycerides |
|  | High-density Lipoprotein Cholesterol |
|  | C-Reactive Protein |
|  | Glycohemoglobin (HbA1c) |
|  | Homeostatic Model Assessment Insulin Resistance (HOMA-IR) |
|  | Platelet Count |
|  | Hemoglobin |
|  | Aspartate aminotransferase (AST) |
|  | Alanine aminotransferase (ALT) |
|  | Total Bilirubin |
|  | Albumin |
|  | Uric Acid |
|  | Creatinine |
|  | Ferritin |

**
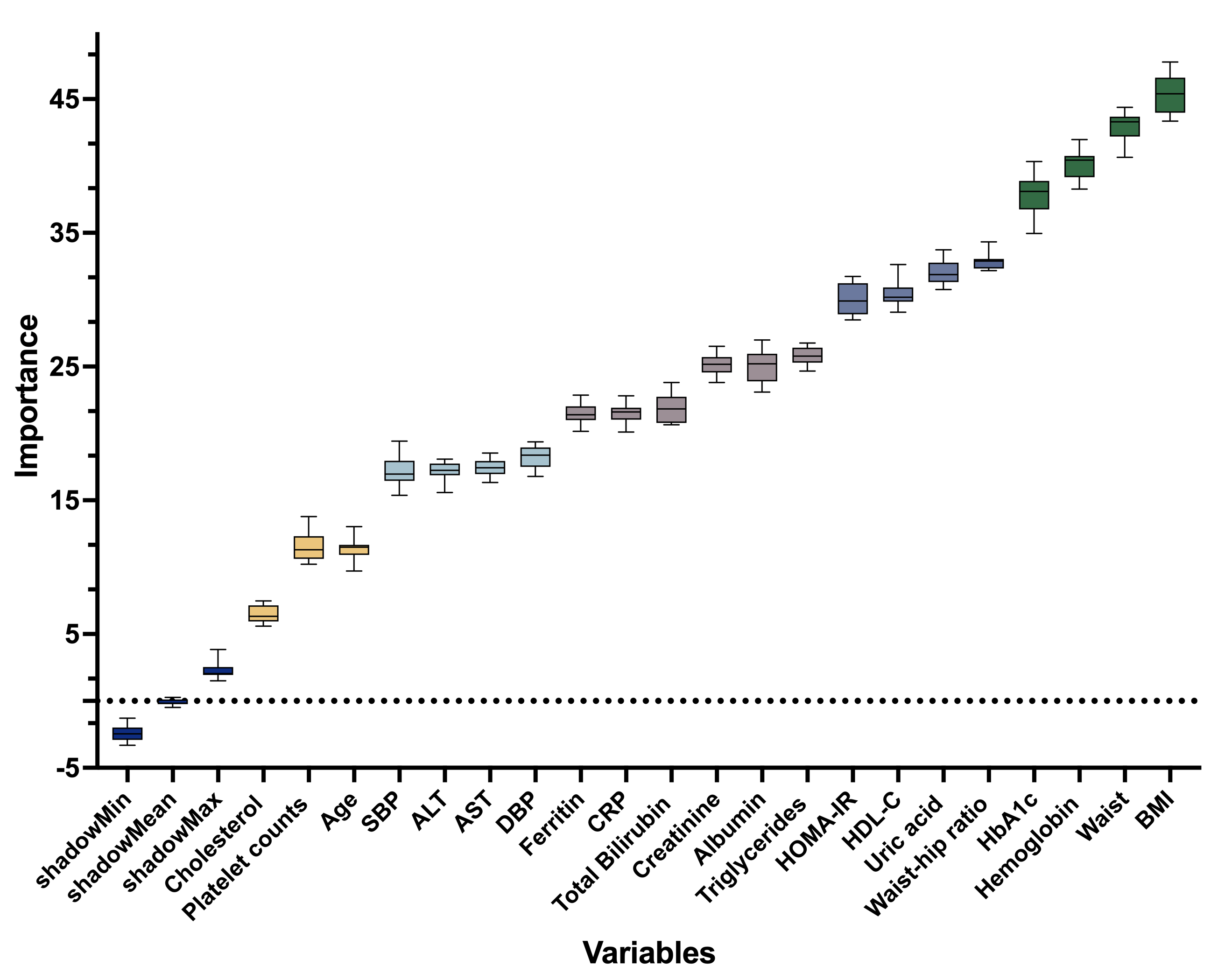
**

**Supplementary Figure 2. Variables importance assessed by Boruta analysis**

Abbreviations: SBP, systolic blood pressure; ALT, Alanine Aminotransferase; AST, Aspartate Aminotransferase; DBP, diastolic blood pressure; CRP, C-reactive protein; HOMA-IR, homeostatic model assessment–insulin resistance; HDL-C high-density lipoprotein cholesterol; BMI, body mass index.

**
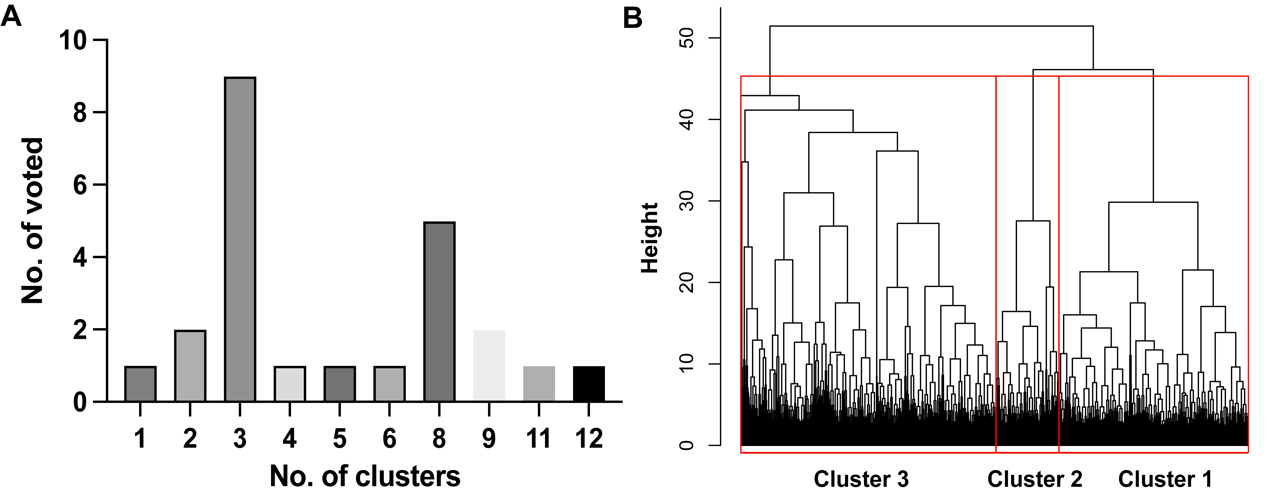
**

**Supplementary Figure 3. The bar plot determines the optimum number of clusters (A) and dendrogram of the final hierarchical clustering model(B) in NHANES 2017-March 2020.**

Wald’s minimum-variance hierarchical clustering method and the bottom-up approach were used. All subjects were clustered into a single final group. At each generation of clusters, samples were merged into larger clusters to minimize the within-cluster sum of squares or maximize the between-cluster sum of squares. With successive clustering, 3 groups became obvious.

**Supplementary Table 1. Baseline Characteristics of the study population in NHANES 2017-March 2020.**

|  | **Overall**  **(n=1082)** | **Cluster 1**  **(n=404)** | **Cluster 2**  **(n=134)** | **Cluster 3**  **(n=544)** | *p* value |
| --- | --- | --- | --- | --- | --- |
| **Age (years)** | 53.2 (15.8) | 51.1 (16.0) | 51.0 (14.1) | 55.2 (15.7) | <0.01 |
| **Female (%)** | 506 (46.8) | 256 (63.4) | 115 (85.8) | 135 (24.8) | <0.01 |
| **Race/ethnicity (%)** |  |  |  |  | <0.01 |
| Non-Hispanic White | 415 (38.4) | 151 (37.4) | 48 (35.8) | 216 (39.7) |  |
| Non-Hispanic Black | 206 (19.0) | 66 (16.3) | 46 (34.3) | 94 (17.3) |  |
| Mexican American | 183 (16.9) | 65 (16.1) | 21 (15.7) | 97 (17.8) |  |
| Other Hispanic | 109 (10.1) | 48 (11.9) | 12 (9.0) | 49 (9.0) |  |
| Non-Hispanic Asian | 117 (10.8) | 52 (12.9) | 1 (0.7) | 64 (11.8) |  |
| Other | 52 (4.8) | 22 (5.4) | 6 (4.5) | 24 (4.4) |  |
| **Current Smoker (%)** | 482 (44.5) | 150 (37.1) | 57 (42.5) | 275 (50.6) | <0.01 |
| **Hypertension (%)** | 623 (57.6) | 182 (45.0) | 85 (63.4) | 356 (65.4) | <0.01 |
| **SBP (mmHg)** | 126.5 (17.9) | 120.9 (14.7) | 122.6 (16.6) | 131.6 (18.8) | <0.01 |
| **DBP (mmHg)** | 76.6 (11.3) | 73.7 (9.6) | 77.5 (11.1) | 78.5 (12.0) | <0.01 |
| **Waist circumference (cm)** | 110.9 (15.0) | 102.5 (10.4) | 127.6 (13.7) | 113.0 (14.0) | <0.01 |
| **Waist-hip ratio** | 1.0 (0.1) | 0.9 (0.1) | 1.0 (0.1) | 1.0 (0.1) | <0.01 |
| **BMI (kg/m^2^)** | 33.7 (7.0) | 30.6 (4.6) | 43.5 (7.4) | 33.6 (6.3) | <0.01 |
| **Diabetes (%)** | 348 (32.2) | 78 (19.3) | 49 (36.6) | 221 (40.6) | <0.01 |
| **Insulin resistance (%)** | 817 (75.5) | 258 (63.9) | 117 (87.3) | 442 (81.2) | <0.01 |
| **HbA1c (%)** | 6.3 (1.4) | 5.8 (0.7) | 6.1 (0.8) | 6.7 (1.8) | <0.01 |
| **Fasting glucose (mg/dL)** | 125.8 (46.9) | 112.4 (23.7) | 117.0 (27.1) | 138.0 (59.0) | <0.01 |
| **Hemoglobin (g/dL)** | 14.3 (1.5) | 13.9 (1.3) | 13.3 (1.3) | 14.8 (1.5) | <0.01 |
| **Platelet counts (1000 cells/uL)** | 245.5 (65.7) | 260.0 (64.4) | 288.9 (73.2) | 224.1 (55.8) | <0.01 |
| **Ferritin, (ng/mL)** | 125.0 [63.5, 229.0] | 104.5 [47.7, 168.0] | 93.2 [49.3, 153.0] | 164.0 [84.9, 298.8] | <0.01 |
| **hs-CRP (mg/L)** | 2.9 [1.3, 5.9] | 2.2 [1.1, 4.3] | 10.3 [5.4, 19.5] | 2.8 [1.3, 5.0] | <0.01 |
| **Cholesterol (mg/dL)** | 186.6 (41.8) | 191.5 (41.3) | 180.9 (31.6) | 184.3 (44.0) | <0.01 |
| **Triglycerides (mg/dL)** | 134.0 [96.0, 184.0] | 122.5 [87.0, 168.0] | 114.0 [90.0, 148.5] | 145.0 [109.8, 209.8] | <0.01 |
| **HDL-C (mg/dL)** | 47.9 (13.4) | 51.6 (15.3) | 52.6 (11.7) | 44.0 (10.9) | <0.01 |
| **Total Bilirubin (mg/dL)** | 0.5 (0.3) | 0.4 (0.2) | 0.4 (0.2) | 0.5 (0.3) | <0.01 |
| **AST (U/L)** | 19.0 [16.0, 25.0] | 19.0 [16.0, 22.0] | 17.0 [14.0, 22.0] | 21.0 [17.0, 28.0] | <0.01 |
| **ALT(U/L)** | 21.0 [16.0, 31.0] | 19.0 [15.0, 25.2] | 18.0 [13.0, 24.8] | 25.0 [17.0, 40.2] | <0.01 |
| **Albumin (g/dL)** | 4.0 (0.3) | 4.0 (0.3) | 3.7 (0.3) | 4.0 (0.3) | <0.01 |
| **Uric acid (mg/dL)** | 5.9 (1.5) | 5.3 (1.1) | 5.7 (1.4) | 6.3 (1.6) | <0.01 |
| **Creatinine (mg/dL)** | 0.9 (0.4) | 0.8 (0.2) | 0.8 (0.2) | 1.0 (0.5) | <0.01 |
| **eGFR (ml/min/1.73m^2^)** | 95.0 (29.0) | 99.0 (25.1) | 95.2 (22.1) | 92.0 (32.7) | <0.01 |
| **Advanced fibrosis (%)** | 56 (5.2) | 5 (1.2) | 13 (9.7) | 38 (7.0) | <0.01 |
| **NASH, %** | 147 (13.6) | 13 (3.2) | 16 (11.9) | 118 (21.7) | <0.01 |

Values were displayed as mean (standard deviation) or median [interquartile range] for continuous variable and count (percentage) for categorical variable.

Abbreviations: NHANES, national health and nutrition examination surveys; SBP, systolic blood pressure; DBP, diastolic blood pressure; BMI, body mass index; HbA1c, glycohemoglobin; hs-CRP, high-sensitivity C-reactive protein; HDL-C high-density lipoprotein cholesterol; AST, Aspartate Aminotransferase; ALT, Alanine Aminotransferase; eGFR, estimated glomerular filtration rate; NASH, nonalcoholic steatohepatitis.
